# Research on Motion Trajectory Correction Method for Wall-Climbing Robots Based on External Visual Localization System

**DOI:** 10.3390/s26030773

**Published:** 2026-01-23

**Authors:** Haolei Ru, Meiping Sheng, Fei Gao, Zhanghao Li, Jiahui Qi, Lei Cheng, Kuo Su, Jiahao Zhang, Jiangjian Xiao

**Affiliations:** 1 School of Marine Science and Technology, Northwestern Polytechnical University, Xi’an 710072, China; ruhl@nimte.ac.cn; 2 Ningbo Institute of Materials Technology and Engineering, Chinese Academy of Sciences, Ningbo 315201, China; gaofei@nimte.ac.cn (F.G.); 2411980017@nbu.edu.cn (Z.L.); qijiahui@nimte.ac.cn (J.Q.); chenglei@nimte.ac.cn (L.C.); sukuo@nimte.ac.cn (K.S.); zhangjiahao@nimte.ac.cn (J.Z.); xiaojj@nimte.ac.cn (J.X.)

**Keywords:** wall-climbing robots, extended Kalman filter, visual positioning system, trajectory correction

## Abstract

To reduce manual operation and enhance the intelligence of the high-altitude maintenance wall-climbing robot during its operation, path planning and autonomous navigation need to be implemented. Due to non-uniform magnetic adhesion between the wall-climbing robot and the steel plate, often caused by variations in steel thickness or surface pitting, the wall-climbing robot may experience motion deviations and deviate from its planned trajectory. In order to obtain the actual deviation from the expected trajectory, it is necessary to accurately locate the wall-climbing robot. This allows for the generation of precise control signals, enabling trajectory correction and ensuring high-precision autonomous navigation. Therefore, this paper proposes an external visual localization system based on a pan–tilt laser tracker unit. The system utilizes a zoom camera to track an AprilTag marker and drives the pan–tilt platform, while a laser rangefinder provides high-accuracy distance measurement. The robot’s three-dimensional (3D) pose is ultimately calculated by fusing the visual and ranging data. However, due to the limited tracking speed of the pan–tilt mechanism relative to the robot’s movement, we introduce an Extended Kalman Filter (EKF) to robustly predict the robot’s true spatial coordinates. The robot’s three-dimensional coordinates are periodically compared with the predefined route coordinates to calculate the deviation. This comparison generates closed-loop control signals for the robot’s movement direction and speed. Finally, based on the LoRa communication protocol, closed-loop control of the robot’s movement direction and speed are achieved through the upper-level computer, ensuring that the robot returns to the predefined track. Extensive comparative experiments demonstrate that the localization system achieves stable localization with an accuracy better than 0.025 m on a 6 m × 2.5 m steel structure surface. Based on this high-precision positioning and motion correction, the robot’s motion deviation is kept within 0.1 m, providing a reliable pose reference for precise motion control and high-reliability operation in complex structural environments.

## 1. Introduction

The increasing demands of modern engineering construction have driven the widespread adoption of wall-climbing robots on large steel structures, such as pressure steel pipes in hydropower stations, giant storage tanks, and ship hulls, typically for tasks like welding, grinding, and non-destructive testing [1,2]. The need for intelligent, precise operations has raised higher requirements for the accurate movement of wall-climbing robots. However, the complex working environments and varying surface conditions often result in motion deviations during path planning and autonomous navigation along the predefined routes. Therefore, it is essential to research high-precision automatic positioning methods to obtain three-dimensional coordinate information and then study motion control strategies and path planning methods based on high-precision positioning data, ensuring that the wall-climbing robot moves strictly according to the planned path.

Existing localisation systems for wall-climbing robots mainly include Inertial Navigation Systems (INS), GPS, Wi-Fi, Bluetooth, UWB, LiDAR, and Vision [3,4]. Existing localization methods can be categorized into two types based on the sensor placement: on-board sensors and external sensors [5]. On-board sensors typically include IMUs, cameras, LiDARs, and wheel odometers. For example, Gu et al. [6] employed visual odometry to achieve global localization without relying on any external infrastructure. Zhang et al. [7] realized information fusion of encoders and IMUs on curved surfaces in low-light environments by observing ground ArUco markers with a fish-eye camera. The ORB-SLAM algorithm proposed by Mur-Artal et al. [8] used ORB features for tracking, localization, and mapping, enhancing stability in complex environments. However, constrained by the lightweight design of wall-climbing robots and the requirements for large steel structure surface operations, they often cannot carry high-computing-power devices or substantial loads. Furthermore, the limited field of view of on-board cameras makes it difficult to continuously keep feature markers within view, restricting the applicability of on-board localization within the camera’s sight range, thus making on-board localization less applicable for the autonomous localization of wall-climbing robots. 

In contrast, external localization methods mainly rely on wireless signal networks or external vision systems. For example, Enjikalayil Abdulkader et al. [9] achieved the localization of a magnetic climbing robot on a ship hull using an ultrasonic beacon system. Li et al. [10] from Zhejiang University remotely identified the wheel position and orientation angle of a wall-climbing robot using an infrared camera together with a Convolutional Neural Network (CNN). Wang et al. [11] integrated a pan–tilt unit, a zoom camera, and a laser rangefinder to perform 3D localization through image scanning and point-cloud registration. Their system achieved a 2D localization error of less than 8 pixels and a spatial error below 0.12 m within a 28 m range, with point-cloud accuracy better than 2.5 mm. Compared to on-board localization, external sensors reduce the robot’s load and energy consumption, and their error does not accumulate over time, resulting in higher overall stability.

However, steel structures strongly absorb electromagnetic signals, which can cause interference or even failure of wireless localization systems [12,13], and high-precision solutions such as UWB often incur substantial cost. Although INS offers strong autonomy and robustness against external disturbances, it suffers from cumulative drift, leading to a degradation of localization accuracy over time. Its internal magnetometers are also severely affected by magnetic interference [14]. Vision-based sensors can effectively mitigate multi-path effects, ensure stable signal transmission and reception, and offer good adaptability to complex operating conditions. However, due to limitations in the field of view and measurement precision, vision sensors are generally suitable only for short-distance, short-range localization [15].

Autonomous navigation of mobile robots mainly involves autonomous localization and path planning strategies [16], with much of the research focusing on ground mobile robots. Luo Kelong et al. [17] proposed a new outdoor positioning and navigation algorithm based on multi-sensor fusion technology. The algorithm collects environmental data through various sensors and uses Bayesian methods to fuse the preliminary data. Subsequently, the Extended Kalman Filter (EKF) algorithm processes the data. By calculating the optimal variance of the output parameters, this algorithm achieves precise positioning of the robotic dog in complex environments. Experimental results show that this method can efficiently identify and avoid obstacles while executing tasks in different scenarios, achieving high recognition accuracy and quickly determining the optimal route. It effectively addresses the challenges of path planning and navigation in complex environments. The elastic band algorithm proposed by Sprunk et al. [18] implements obstacle avoidance in global path planning by expanding obstacles based on the mobile robot’s inscribed circle radius. The mobile robot locates itself and adjusts its pose when the distance to the nearest obstacle is smaller than the circumscribed circle radius, thus avoiding collisions. Additionally, reinforcement learning can be applied in autonomous navigation. By training agents and using environmental observations and goal localization information, the robot can react according to learned strategies, achieving optimal path planning and navigation strategies [19]. In indoor environments, Park et al. [20] utilized ultra-wideband (UWB) positioning technology to achieve precise localization of the robot, while using Building Information Modelling (BIM) as the environmental map for path planning, thus enabling autonomous navigation. In addition, LiDAR technology has been widely used for localization, mapping, and path planning due to its minimal environmental interference and the ability to provide precise depth information [21]. In China, Wu Weiguo, Ding Rui, and others [22] proposed a global localization method based on a laser emitter board to address the backend overload problem in laser SLAM algorithms. This localization method offers high-precision performance and effectively meets the accuracy requirements for robot navigation. For wheeled unmanned ground vehicles (UGVs), under uncertain slipping conditions such as wet surfaces, tracking accuracy often decreases, and issues such as high-speed command saturation may occur. Conceicao A. G. S. et al. [23] proposed a trajectory correction method under slip conditions by combining feedback linearization-based control laws to adjust UGV speed and angular velocity. Dambly. V. et al. [24] used a fifth-order Hermite spline discrete trajectory and iterative relocation nodes, combined with an error window weighting strategy and key area node supplementation, to realize offline iterative correction of the trajectory, thereby compensating for static and dynamic deviations in robot processing. High-precision positioning of mobile robots can meet the accuracy requirements of autonomous navigation and trajectory correction processes. Current research on trajectory correction and autonomous positioning navigation for mobile robots mainly focuses on designing controllers based on information obtained from the robot’s own sensors to achieve position correction. However, due to the complex environment in which wall-climbing robots operate, sensor sensitivity is often lacking. Therefore, traditional positioning and correction methods are insufficient to meet the closed-loop control requirements of wall-climbing robots.

Therefore, this paper proposes a wall-climbing robot deviation correction system based on external visual recognition and localization. The system fully leverages the stability of external sensor-based positioning and the powerful computing and control capabilities of the upper-level computer. To address the issues of tracking deviation and laser-projection outliers, the system introduces Extended Kalman Filtering (EKF) to enhance the robustness of the positioning algorithm and calculate the high-precision three-dimensional coordinates of the wall-climbing robot. The upper-level computer of the trajectory correction system collects and compares the predefined path and actual coordinates, generating control signals for the robot’s movement direction and speed. This allows for real-time control and motion correction of the wall-climbing robot, ensuring that it follows the planned path and adheres strictly to the predefined trajectory. Based on the positioning coordinates, the robot’s motion control is displayed, and finally, the system is validated through multiple sets of positioning simulations and positioning correction experiments. The results demonstrate that the system can achieve high-precision positioning of the wall-climbing robot, and this positioning information can effectively guide the robot’s movement, enabling highly accurate path planning.

The remainder of this paper is organized as follows: Section 2 introduces the external visual pan–tilt positioning system based on the Extended Kalman Filter (EKF) method, including the composition and principles. Section 3 focuses on the real-time trajectory correction method for the wall-climbing robot movement based on the high-precision positioning pan–tilt, achieving path planning and motion along the planned path. Section 4.1 discusses the EKF simulation of the wall-climbing robot’s positioning pan–tilt, validating the positioning performance of EKF. Section 4.2 compares the experimental results of four filter algorithms for wall-climbing robot positioning prediction, as well as various motion control correction strategies based on EKF positioning prediction information. Section 5 provides a discussion of the results.

## 2. Visual Positioning System Based on Extended Kalman Filter Algorithm

### 2.1. Introduction to Motion-Based External Visual Positioning System

**Figure 1:**
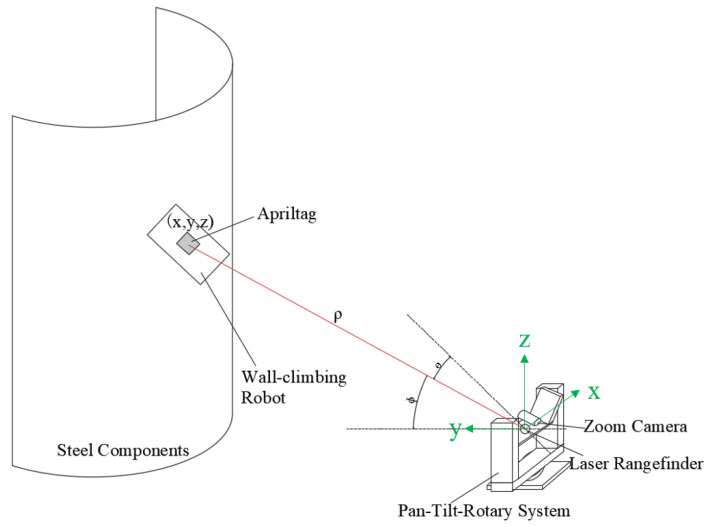
Schematic of the positioning system.

The real-time localization system, based on pan–tilt tracking and visual positioning fusion, is illustrated in Figure 1. This system consists of two primary components: (1) a wall-climbing robot equipped with an AprilTag marker, and (2) an external vision device consisting of a two-axis rotational pan–tilt mechanism, a laser rangefinder, and a zoom camera.

The Zoom camera and the AprilTag form the fiducial (feature) tracking module of the system. The AprilTag is mounted on the exterior surface of the wall-climbing robot, while the camera is fixed on the pan–tilt unit. Initially, the camera uses a short focal length to obtain a wide field of view, allowing it to capture the entire workspace target and estimate the approximate position of the target. Subsequently, the focal length is then increased to narrow the field of view, thereby enhancing image resolution and ensuring more robust AprilTag detection. AprilTag employs a unique encoding scheme and image-processing algorithm [19], providing strong robustness against disturbances caused by complex surface textures—such as rust and paint on steel structures—as well as varying lighting conditions. This ensures stable visual detection accuracy and recognition efficiency. Once the AprilTag is detected, the camera calculates the offset *Δ* between the tag’s centre and the image centre, which is then fed back to control the platform’s pan and tilt motions. The pan–tilt unit accordingly adjusts the orientation of the zoom camera and laser rangefinder to minimize *Δ*, achieving continuous tracking of the AprilTag.

The two-axis pan–tilt unit, together with the laser rangefinder, forms the data-measurement module, which acquires the pan angle *θ*, tilt angle *φ*, and the distance *ρ* between the camera lens and the Apriltag during tracking. The horizontal rotation axis of the pan–tilt must offer a range exceeding 180°, while the vertical axis must support a range from −30° to +60°. Since the initial positioning coordinates are generated in the pan–tilt coordinate frame, the rotational angles are obtained from the encoders of the horizontal and vertical axes. Combined with the distance measured by the laser rangefinder, these angles yield the spherical coordinates of the laser tracking point. Using Equation (1), these spherical coordinates are converted into Cartesian coordinates, with the origin located at the laser rangefinder. In this coordinate system, the z-axis is oriented vertically upward from the ground, and the x-axis aligns with the optical axis of the laser rangefinder, thereby providing the target’s spatial localization information. To minimize positioning errors caused by misalignment between the pan–tilt and the laser rangefinder, the laser emission point must be precisely aligned with the intersection of the pan–tilt’s two rotational axes.

(1) $$\left\{\begin{array}{l} X=\rho \sin\phi \cos\theta \\ Y=\rho \sin\theta \\ Z=\rho \cos\phi \cos\theta \end{array}\right.$$

During pan–tilt tracking of the AprilTag, the agile motion of the wall-climbing robot and the operational environment make it difficult for the pan–tilt to adjust its speed adaptively. Consequently, the centre of the camera image does not align precisely with the centre of the detected target. Additionally, because the laser rangefinder acquires measurements at a fixed point on the robot, its readings may not consistently correspond to a fixed point on the robot. As a result, the computed 3D coordinates do not represent a stable point on the robot, producing outliers in the fitted motion trajectory. These erroneous points are defined as “noise”. Notably, the magnitude and frequency of these noise points increase with more abrupt or larger changes in the robot’s velocity, thereby reducing the accuracy of the robot’s motion location.

To address this issue, it is necessary to apply a filter algorithm to smooth the nonlinear, effectively reducing or eliminating the impact of outliers on 3D coordinate measurements. Taking into account both the requirements of processing effectiveness and speed of the positioning, we apply the Extended Kalman Filter algorithm (EKF) to estimate the position of the wall-climbing robot using the external Visual Positioning System. This approach can produce a smoother motion trajectory and provide more accurate 3D localization for the wall-climbing robot.

Assuming the wall-climbing robot undergoes linear motion, its state equation for linear motion is expressed as $s=s_{t-1}+{\dot{s}}_{t-1}\Delta t+(u\Delta t^{2}/2)$, where *Δt* denotes the time interval between the previous and current time steps, during which the robot is assumed to move at a constant velocity. The linear motion equation at the next time step $s_{t}^{\prime }$ is $s_{t}^{\prime }=Hs_{t}+v_{t}$. The observation noise *v*_t_ arises from the pan–tilt–rotary system’s inability to perfectly track the robot, resulting in measurements contaminated by tracking-failure noise. Based on this measurement, the linear filter predicts the robot’s position at the next time step using the following state equation,

(2) $$G_{t}=G_{t-1}+F_{t-1}^{T}V_{t-1}+A_{t-1}H_{t-1}+\omega_{t-1}$$

Thereinto,

(3) $$G_{t}=\left[\begin{array}{c} x_{t}\\ y_{t}\\ z_{t} \end{array}\right],V_{t-1}=\left[\begin{array}{c} v_{t}^{x}\\ v_{t}^{y}\\ v_{t}^{z} \end{array}\right],F_{t-1}=\left[\begin{array}{c} \Delta t\\ \Delta t\\ \Delta t \end{array}\right],A_{t-1}=\left[\begin{array}{c} a_{t}^{x}\\ a_{t}^{y}\\ a_{t}^{z} \end{array}\right],H_{t-1}=\left[\begin{array}{c} \Delta t^{2}/2\\ \Delta t^{2}/2\\ \Delta t^{2}/2 \end{array}\right]$$

In the formula, *G_t_* is the three-dimensional coordinate estimator, *V*_*t*−1_ denotes the velocity state at time *t*−1, and *A*_t−1_ denotes the acceleration state value at time *t*−1. At the same time, *F*_*t*−1_ and *A*_*t*−1_ serve as the state transition coefficients at time *t*−1, and *w*_*t*−1_ is the process noise vector.

The pan–tilt–rotary (PTR) positioning system provides the observation model of the wall-climbing robot.

(4) $$G_{t}=f(\rho_{t},\sin\phi_{t},\cos\phi_{t})+U$$

Thereinto,

(5) $$f=\left[\begin{array}{l} \rho_{t}\sin\phi_{t}\cos\theta_{t}\\ \rho_{t}\sin\phi_{t}\\ \rho_{t}\cos\phi_{t}\cos\theta_{t} \end{array}\right],U=\left[\begin{array}{c} u_{x}\\ u_{y}\\ u_{z} \end{array}\right]$$

The *f* specifies the specific conversion formula for the observed value; the deviation noise *U* of the positioning gimbal is only related to *ρ*, meaning that the positioning gimbal cannot successfully catch up with the wall-climbing machine.

### 2.2. Coordinate Estimation of Positioning Based on Extended Kalman Filter

For nonlinear systems calculating 3D coordinates from gimbal laser-marked sampling data, the Kalman Filter algorithm is not suitable; the EKF algorithm is commonly used. Predicting the precise position coordinates of the wall-climbing robot using the EKF algorithm yields accurate position coordinates. Following the Standard Kalman Filter procedure, the motion state prediction and gimbal positioning update algorithms for the wall-climbing robot are summarized as follows:

➀ The motion system states of the wall-climbing robot are position and velocity, which are typically defined as

(6) $$X_{k}=\left[\begin{array}{c} x_{k}\\ y_{k}\\ z_{k}\\ v_{x}\\ v_{y}\\ v_{z} \end{array}\right],$$

where *x_k_*, *y_k_*, and *z*_k_ represent the position of the wall-climbing robot, and *v*_x_, *v_y_*, and *v_z_* represent the velocity of the robot.

➁ Motion state prediction model, given $G_{k}=G_{k-1}+F_{k-1}^{T}V_{k-1}+A_{k-1}H_{k-1}+\omega_{k-1}$, its EKF nonlinear form is

(7) $$X_{k}=f(X_{k-1},u_{k-1})+w_{k-1},$$

where *f(.)* is the state transition function, *u*_*k*−1_ is the control input (velocity *V*_*k*−1_, acceleration *A*_*k*−1_), and *w*_*k*−1_ is the process noise; it is typically the noise with covariance (Q), which describes the uncertainty in the system model. The equations are linearized, and the Jacobian matrix *F_k_* is calculated, which is the partial derivative of the state transition function *f(.)* with respect to the state *X*_*k*−1_.

(8) $$F_{k}=\frac{\partial f}{\partial X}|X_{k-1}=\left[\begin{array}{cc} I_{3\times 3} & \Delta tI_{3\times 3}\\ 0 & I_{3\times 3} \end{array}\right],$$

where Δt is the time interval.

➂ Prediction: Make predictions based on the current state and control inputs.

(9) $${\hat{X}}_{k}^{+}=f({\hat{X}}_{k-1}^{-},u_{k-1})$$

The prediction of covariance is as follows

(10) $$P_{k}^{-}=F_{k}P_{k-1}^{+}F_{k}^{T}+Q$$

➃ Observation model: The EKF standard for the observation equation is

(11) $$Z_{k}=h(X_{k})+v_{k},$$

where *h(.)* is the observation function, and *v_k_* is the observation noise, which is only caused by the gimbal tracking delay. The observation function only involves the robot’s position, so it can be written as

(12) $$h(X_{k})=f(\rho_{r},\sin\phi_{r},\cos\phi_{r})+U$$

➄ Jacobian observation: For the observation function *h(X_k_)*, where the partial derivative of the Jacobian matrix *H*_k_ with respect to the observation function is

(13) $$H_{k}=\frac{\partial h}{\partial X}{|}_{X_{k}}=[I_{3\times 3},0_{3\times 3}]$$

➅ Kalman gain:

(14) $$K_{k}=P_{k}^{-}H_{k}^{T}{\left(H_{k}P_{k}^{-}H_{k}^{T}+R\right)}^{-1},$$

where *R* is the covariance matrix of the observation noise, caused by the gimbal tracking delay, so the state correction is

(15) $${\hat{X}}_{k}^{+}={\hat{X}}_{k}^{-}+K_{k}(Z_{k}-{\hat{Z}}_{k}),$$

where ${\hat{Z}}_{k}=h({\hat{X}}_{k}^{-})$ is the observed value calculated based on the predicted state.

The covariance is updated as follows:

(16) $$P_{k}^{+}=(I-K_{k}H_{k})P_{k}^{-}.$$

## 3. Automatic Correction Method of the Wall-Climbing Robot Based on Visual Positioning System

**Figure 2:**
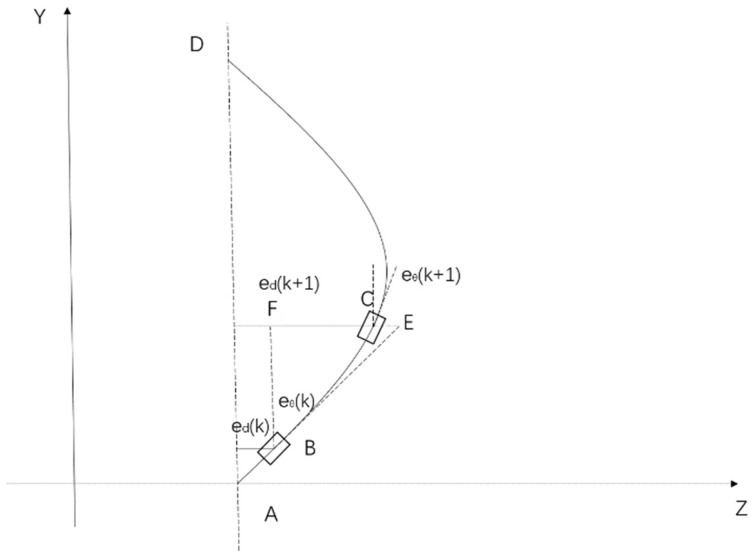
Deviation of the movement trajectory of the wall-climbing robot.

Due to inherent structural errors and external environmental disturbances, wall-climbing robots inevitably develop angular deviations (*e_θ_*) and distance deviations (*e_d_*) during operation. The proposed pan–tilt–rotary positioning system provides precise positioning and closed-loop control to mitigate these deviations. In the formulation of the motion model, the desired trajectory of the robot is defined as a straight-line AD, with a central velocity of *V*, a gimbal sampling time (control cycle) of *T_s_*, and a body centre angle *ω* during curved movement, as illustrated in Figure 2.

During robot movement, trajectory deviation arises. The perpendicular distance from the robot’s centre point to the target line defines the distance deviation (*e_d_*), whereas the angle deviation (*e_θ_*) is the angle between the robot’s forward extension line BE and the target trajectory AD. To eliminate both angular and distance deviations during correction control, the robot’s actual trajectory follows a curve path that progressively approaches the desired trajectory over time. Let point B denote the robot’s position at time *k*. According to the model’s prediction principles, point C is expected to be the robot’s desired position at time *k* + 1. Thus, *e_θ_*(*k*) and *e_d_*(*k*) denote the angular and distance deviations at time *k*, while *e_θ_*(*k* + *1*) and *e_d_*(*k* + *1*) represent the corresponding deviations at time *k* + 1.

The relationship between the robot’s angular velocity and time within the current control cycle can be used to determine the angular deviation between the robot at times *k* and *k* + 1.

(17) $$e_{\theta }(k+1)=e_{\theta }(k)+\varpi (k)T_{S}$$

As shown in Figure 2, the relationship between the distance deviation at time *k* and time *k* + 1 can be derived as follows:

(18) $$e_{d}(k+1)=e_{d}(k)+EF-CE$$

To obtain the distance deviation relationship, it is necessary to compute the segments EF and CE. Because the time interval between two adjacent sampling cycles is very short, the range of angular velocity variation is negligible. Therefore, the actual trajectory curve BC between time *k* and *k* + 1 can be approximated as a straight-line BE, which allows us to derive the straight-line segments BF and BE.

(19) $$BC=T_{S}V,$$

(20) $$BF=T_{S}V\tan e_{\theta }(k),$$

(21) $$BE=\frac{BF}{\cos e_{\theta }(k)}.$$

It can be obtained from the relationship of the trajectories. The relationship between CE and EF can be derived based on the angles ∠EBC and ∠EBF.

(22) $$\frac{CE}{EF}=\frac{\varpi (k)T_{s}}{e_{\theta }(k)},$$

(23) $$CE=\frac{T_{S}^{2}V\varpi (k)\tan e_{\theta }(k)}{e_{\theta }(k)}.$$

According to the above, we can obtain the distance deviation relationship between *k* and *k* + 1,

(24) $$e_{d}(k+1)=e_{d}(k)+T_{S}V\tan e_{\theta }(k)-\frac{T_{S}^{2}V\varpi (k)\tan e_{\theta }(k)}{e_{\theta }(k)}$$

To facilitate subsequent solution, the above equation needs to be linearized and simplified. This form corresponds to a small pose deviation, so the angular deviation is relatively minor. When the angle is small, it can be approximated as. Thus, we can conclude that

(25) $$e_{d}(k+1)=e_{d}(k)+T_{S}V\tan e_{\theta }(k)-T_{S}^{2}V\varpi (k)$$

In conclusion, the kinematic model for the slight pose deviation of angle and distance at time *k* + 1 is

(26) $$\left\{\begin{array}{l} e_{\theta }(k+1)=e_{\theta }(k)+\varpi (k)T\\ e_{d}(k+1)=e_{d}(k)+T_{S}Ve_{\theta }(k)-T_{S}^{2}V\varpi {\left(k\right)}_{s} \end{array}\right.$$

The high-precision prediction of the three-dimensional coordinates *X*(*x*,*y*,*z*) of the climbing robot in the gimbal positioning system based on the Extended Kalman Filter (EKF) algorithm is compared with the predetermined trajectory target X*(x*,y*,z*) to obtain the difference $X-X^{*}$, to obtain the angular deviation *(e_θ_*) and distance deviation (*e_d_*).

(27) $$X_{k+1}-{X_{k+1}}^{*}=e_{d}(k+1)=X_{k}-{X_{k}}^{*}+T_{S}Ve_{\theta }(k)-T_{S}^{2}V\varpi (k).$$

The positioning gimbal monitors the robot’s movement, specifically tracking its deviation from the desired path. During operation, the robot primarily controls its wall-climbing direction through steering servo mechanisms. The host computer transmits control signals via LoRa communication to adjust the steering wheel’s angle, thereby regulating the robot’s trajectory. By adjusting the angular velocity *w*(*k*), the host computer ensures the deviation error approaches zero.

**Figure 3:**
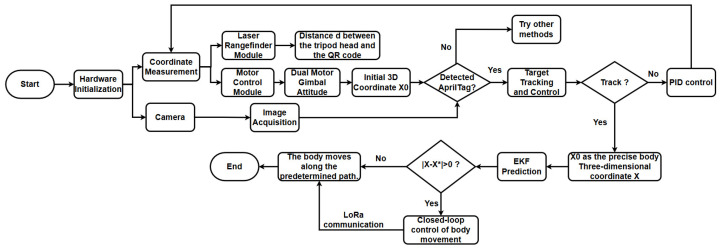
Automatic deviation correction technology route of the wall-climbing robot based on external visual localization.

When the deviation is non-zero, indicating that the robot’s trajectory has strayed from the preset path, the host computer sends reverse control signals via LoRa communication. This establishes a closed-loop control system that guides the robot’s movement to correct its path and follow the predetermined route. The technical process is illustrated in Figure 3.

## 4. Performance Analysis and Validation

### 4.1. Simulation Test

To evaluate the performance of the proposed smooth robust EKF algorithm, a 3D nonlinear motion model for wall-climbing robots was developed in MATLAB 2020b, and the UKF and PF were introduced for verification and comparison. The simulation environment featured a 900 m × 900 m×900 m movement range with 0.2 Hz outliers distributed across three dimensions, where the outlier noise was modelled as $R_{outliner}=diag([0.2^{2},0.2^{2},0.2^{2}])$. Given the three-dimensional observation, the Mahalanobis distance is $d_{k}^{2}$. The data follows a chi-square distribution with three degrees of freedom, where a threshold of nine was chosen to correspond to a high confidence interval. To evaluate the filters’ performance under nonlinear motion, a predefined 3D trajectory combining straight-line and turning motions was designed, assuming the wall-climbing robot moves at constant speed during both phases. The simulation employed a time-segmented trajectory planning method, dividing the motion into three distinct phases: straight-line movement, turning movement, and straight-line movement. This method ensures that the motion model accurately simulates the robot’s movement along the predefined trajectory, providing a fair and controllable benchmark for evaluating the filter. Although this method simplifies real-time control input modelling, it effectively captures the essence of nonlinear motion, sufficiently validating the proposed filtering algorithm. Under these simulation conditions, in addition to the EKF proposed in this paper, we also introduced Unscented Kalman Filter (UKF) and Standard Particle Filter (PF) algorithms to filter and predict the motion trajectory.

**Figure 4:**
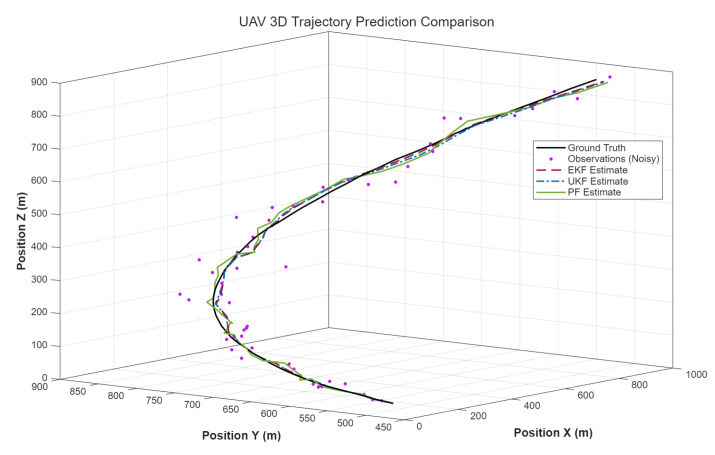
Comparison of simulated trajectories.

**Figure 5:**
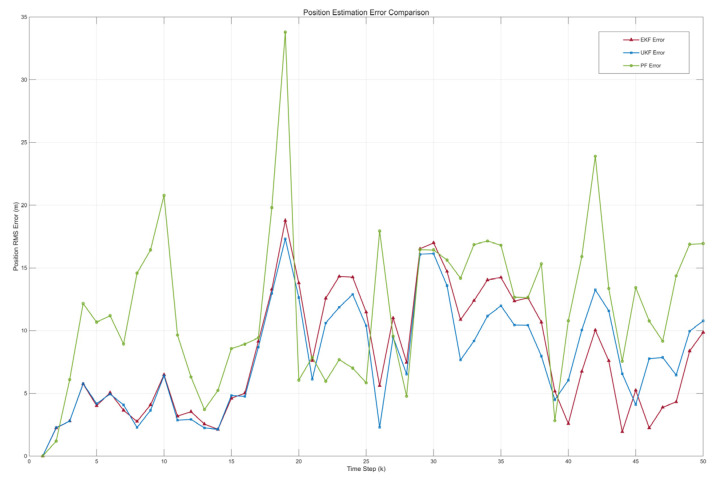
Comparison of instantaneous position errors.

Under the influence of noise, the motion trajectory will have obvious outliers, deviating from the true trajectory. Therefore, it is necessary to use three filtering algorithms, EKF, UKF, and PF, to predict and estimate the motion trajectory and correct the observed outliers. As shown in Figure 4, comparing the four trajectories, it can be seen that even under the interference of outlier random noise. After being processed by filtering algorithms, the motion trajectory of the wall-climbing robot can still closely match the true trajectory. Furthermore, combining the instantaneous position error comparison in Figure 5 and the simulation error comparison results of each algorithm in Table 1, the EKF, UKF, and PF have a significant correction effect on the observation error. The prediction correction error of the EKF and UKF is significantly smaller than that of the PF. The PF, based on the Monte Carlo method, is a non-parametric filtering technique that is more suitable for filtering and prediction in high-dimensional, complex nonlinear systems. For simple motion in nonlinear discrete systems, the optimization performance of the two algorithms is not significantly different. But in terms of computation time, the EKF has a significant advantage over the UKF. This is because the EKF does not require selecting a set of “sigma points” during the computation, resulting in a much lower computational complexity compared to the UKF. Based on the above simulation and analysis results, it can be seen that the EKF can predict and correct outlier observation points, making the predicted points closer to the actual motion trajectory of the wall-climbing robot. It can eliminate the deviation of the three-dimensional coordinate values of the positioning caused by the movement delay of the observation gimbal and has strong positioning accuracy and anti-interference ability. It is more adaptable to the needs of high-frequency prediction compensation and meets the high-precision centimetre-level positioning requirements of the wall-climbing robot.

### 4.2. Experiments and Discussion

#### 4.2.1. EKF Algorithm Positioning Optimization Experiment

**Figure 6:**
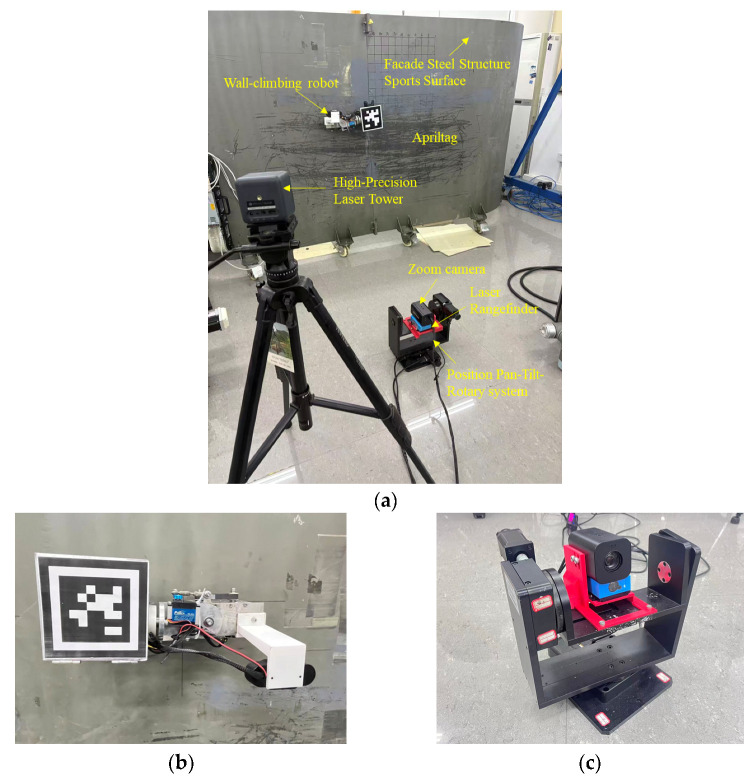
Experimental scenario of the wall-climbing robot: (**a**) experimental system composition; (**b**) the wall-climbing robot; (**c**) pan–tilt–rotary localization system.

The experimental scenario for the comparative positioning system is shown in Figure 6. The wall-climbing robot moves on the surface of a steel plate component, with dimensions of 0.38 m (length) × 0.11 m (width) × 0.17 m (height). The robot employs a magnetic adhesion two-wheel drive structure. A 0.12 m × 0.12 m Apriltag code is fixed to the upper surface of the robot as a marker for tracking. Camera positioning uses a PNP algorithm to detect the Apriltag code for tracking and positioning. A zoom camera and a laser rangefinder are fixed on the central axis of the positioning gimbal in the experiment. The gimbal has a repeatability of 0.01°, an absolute positioning accuracy of 0.03°, and a maximum speed of 15°/*s*. The laser rangefinder has a ranging range of 0.03–100 m, an accuracy of ±0.5 mm, and a maximum sampling frequency of 20 Hz. The zoom camera is located above the laser rangefinder, with a variable focal length of 100–500 mm, an image resolution of 1920 × 1080, and 60 fps. A positioning gimbal drives a zoom camera and a laser rangefinder to rotate, positioned 2 metres away from the steel plate surface. To compare the positioning accuracy of the gimbal system, the true 3D coordinates of the wall-climbing robot are needed. A laser tower is used to obtain the robot’s high-precision 3D coordinates as a reference. The gimbal positioning system acquires the observed values and uses a filtering algorithm to predict the 3D coordinates of the wall-climbing robot. The robot performs a uniform turning motion at a preset speed of 0.1 m/s.

**Figure 7:**
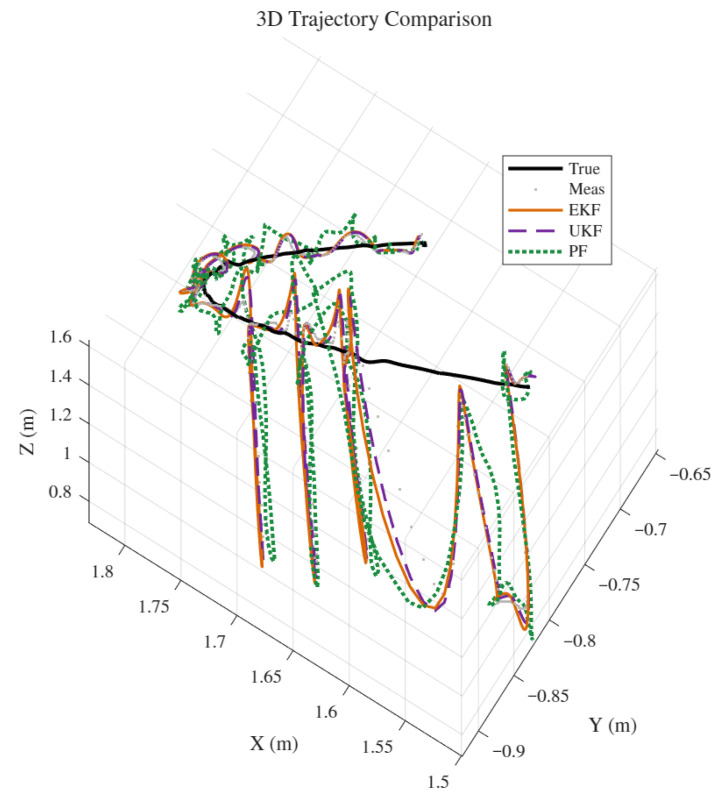
Comparison of robot motion trajectories.

As shown in Figure 7, a visual comparison is presented between the actual trajectory of the wall-climbing robot under uniform turning motion and the measured trajectory processed by EKF, UKF, and PF, as well as the unfiltered trajectory. Experimental verification shows that when significant outlier noise appears in the measurements from the positioning gimbal, all three algorithms can correct the observed trajectory, making it as close as possible to the actual trajectory. Furthermore, the EKF and UKF demonstrate superior correction performance compared to the PF, avoiding erratic trajectory drift. From a subjective visual perspective, the EKF and UKF exhibit strong anti-interference capabilities and effectiveness in handling outlier noise in the tracking trajectory. To further analyze and compare the performance of the EKF and UKF, an objective and quantitative analysis of the correction error and instantaneous computation time of the two filtering algorithms is needed.

**Figure 8:**
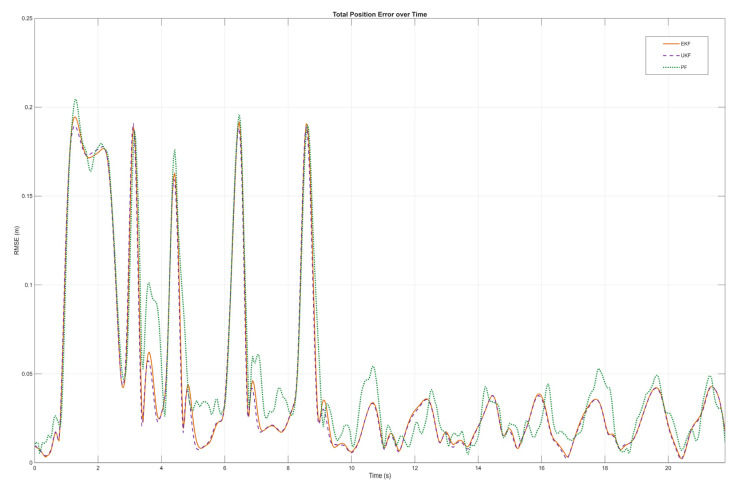
Comparison of instantaneous error.

As shown in Figure 8, the instantaneous errors of the three filtering algorithms are analyzed throughout the process, while Table 2 lists the total root mean square errors (RMSE) of the measured values and the EKF, the RMSE of each axis (*XYZ*), and the average time per step. As can be seen from Figure 8 and Table 2, for different degrees of outliers in the measured trajectory, the EKF, UKF, and PF exhibit good adaptive capabilities and maintain high positioning stability. Table 2 shows that the RMSE for EKF and UFK are 0.0675 m and 0.0667 m, respectively, which are better than that of PF (0.0717 m). This indicates that the filtering effects of EKF and UKF are superior to those of PF, and the difference in their processing effects is not significant, further verifying the above conclusion. Comparing the instantaneous time of each step between EKF and UKF, EKF’s processing time is 0.032 ms, which is shorter than UKF’s. Considering all factors, the EKF is more suitable for the real-time high-frequency processing requirements of motion trajectories. The EKF has not only excellent effects on motion positioning correction but also meets the real-time positioning requirements in terms of filtering processing rate.

#### 4.2.2. Automatic Deviation Correction Experiment of Wall-Climbing Robot Based on External Visual Recognition and Positioning

**Figure 9:**
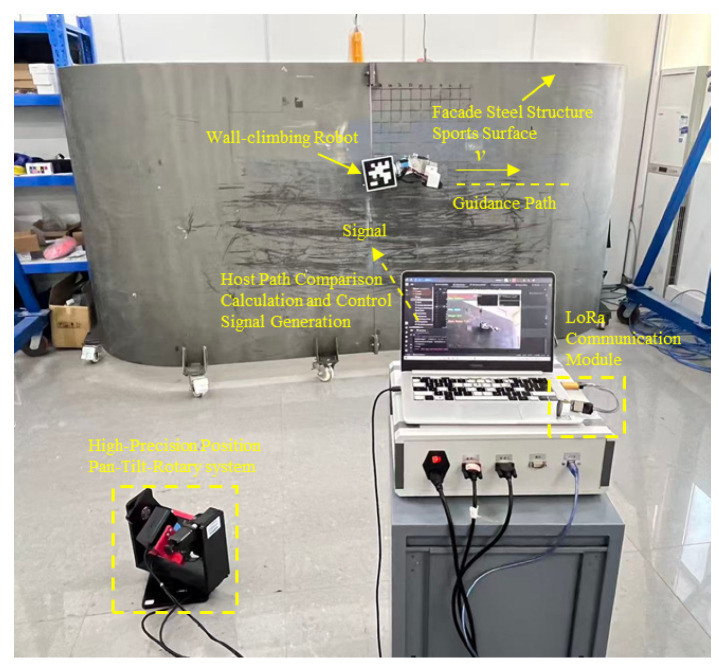
Motion correction process.

**Figure 10:**
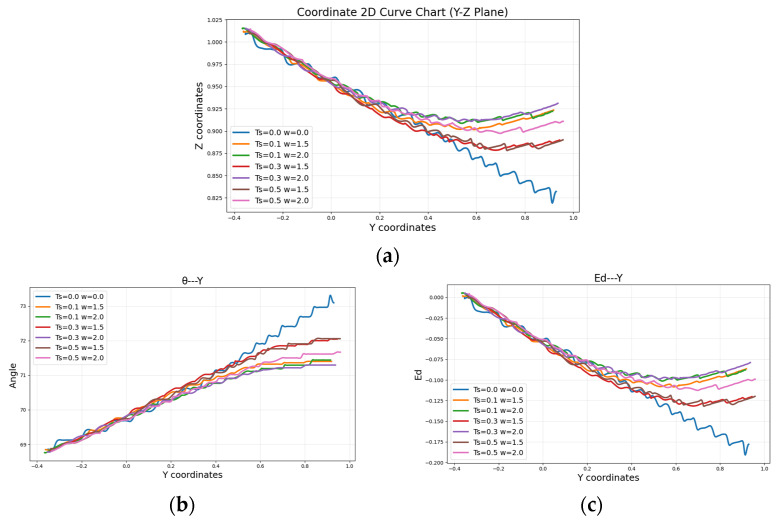
Comparison of motion trajectories: (**a**) Y-Z coordinate curve; (**b**) Y-Angle coordinate curve; (**c**) Y-Ed coordinate curve.

To verify the closed-loop control system of the pan–tilt–rotary positioning system for the wall-climbing robot’s movement, a constant speed *v*_0_ = 0.3 m/s to the left in the horizontal direction is commanded to test its performance without external correction algorithms. The high-precision pan–tilt–rotary positioning system locates 2 metres away from the robot, tracking and positioning the robot’s three-dimensional coordinates. At each sampling period *T*, the upper computer calculates the difference between the positioning coordinates and the predetermined coordinates as *Δ*. Based on this deviation, the inverse control signal is generated. Through the LoRa communication module, the movement of the wall-climbing robot is closed-loop controlled, ensuring it returns to the predetermined track during movement, as shown in Figure 9. Through comparative experiments, the movement trajectories of the wall-climbing robot operating without a positioning correction system were compared with those under external closed-loop positioning correction control. Different sampling positioning cycles *T* are set to 0.1 s, 0.3 s, and 0.5 s, with reverse control signal coefficient *a* set to 1.5 and 2.0 for the pan–tilt head. When the pan–tilt head is not in position and not performing corrections, both the sampling cycle and control signal coefficient *a* are set to 0. For the same direction and distance of movement, the trajectory diagrams of the wall-climbing robot under various pan–tilt head sampling positioning cycles and reverse control signal intensities are shown in Figure 10.

From the graph, it can be observed that when the pan–tilt system does not perform positioning correction, as the y-value continuously increases, the vertical distance from the coordinate axis becomes significantly larger. When the horizontal movement distance reaches 0.95 m, the maximum deviation from the horizontal axis reaches 0.19 m. This deviation is primarily due to the inherent errors in the wall-climbing robot’s structure and the friction in the environment, causing increasing deviation from the planned motion path. Additionally, the smaller the system’s sampling period, the smaller the robot’s deviation from the horizontal axis. As the control signal increases, the deviation from the horizontal axis decreases, and the maximum deviation is reduced to within 0.12 m. When t equals 0.1 s, and *a* equals 2.0, the distance deviation (*e_d_*) from the coordinate axis is reduced to 0.1 m. The pan–tilt positioning correction system can effectively correct the robot’s deviation, bringing it back to the planned path. The shorter the sampling period (*t*), the larger the control signal (*a*). In a shorter control time period, the pan–tilt system can make timely adjustments to the robot’s motion. A larger control gain allows for a greater adjustment angle, ensuring that the wall-climbing robot frequently compares and adjusts to the planned track. This enables the robot to approach the planned path more quickly. The closer the robot’s motion is to the initial position, the stronger the pan–tilt correction effects.

## 5. Conclusions

In summary, the gimbal positioning system based on a camera and laser rangefinder can acquire the 3D coordinates of the wall-climbing robot. To eliminate positioning inaccuracies caused by the gimbal failing to keep up with the robot’s movement, and to balance the positioning correction time, an Extended Kalman Filter (EKF) algorithm is used for prediction and correction, achieving centimetre-level positioning of the wall-climbing robot. Based on this positioning information, the robot’s movement is compared with the expected trajectory in real time, triggering control enhancement signals to guide the robot’s corrective movement and ensure that the movement conforms to the expected trajectory. For complex and variable steel structure surfaces, the shorter the sampling period of this positioning closed-loop control system is, the faster the wall-climbing robot can respond, achieving faster trajectory correction and better distance deviation control. In the future, it can be applied to wall-climbing robots operating at heights, ensuring that the robot’s movement conforms to the pre-set trajectory. However, in actual outdoor industrial environments, ambient light, dust, and vibration can significantly affect the stability and accuracy of the positioning guidance system. Therefore, further research on the accuracy of visual recognition and detection is considered for future development.

## Figures and Tables

**Table 1:** RMSE of different algorithms in simulation (unit: m).

Algorithm	X-Axle RMSE	Y-Axle RMSE	Z-Axle RMSE	Overall RMSE
observed value	0.0434	0.0430	0.0181	0.0637
EKF	0.0446	0.0444	0.0187	0.0656
UKF	0.0443	0.0438	0.0186	0.0650
PF	0.0487	0.0486	0.0229	0.0725

**Table 2:** Positioning performance of different algorithms (RMSE unit: m, Average time unit: ms).

Category	Overall RMSE	X-Axle RMSE	Y-Axle RMSE	Z-Axle RMSE	Average Time per Step
EKF	0.0675	0.0449	0.0466	0.0193	0.018
UKF	0.0667	0.0447	0.0457	0.0191	0.050
PF	0.0717	0.0477	0.0476	0.0245	1.059

## Data Availability

The original contributions presented in this study are included in the article. Further inquiries can be directed to the first author and the corresponding author.
